# Association between rs2431697 T allele on 5q33.3 and systemic lupus erythematosus: case-control study and meta-analysis

**DOI:** 10.1007/s10067-015-3045-4

**Published:** 2015-08-07

**Authors:** Zhao-Ming Tang, Ping Wang, Pan-Pan Chang, Tony Hasahya, Hui Xing, Jin-Ping Wang, Li-Hua Hu

**Affiliations:** Department of Laboratory Medicine, Union Hospital, Tongji Medical College, Huazhong University of Science and Technology, Jiefang Da Dao 1277#, Wuhan, 430022 China; Central Laboratory, Union Hospital, Tongji Medical College, Huazhong University of Science and Technology, Wuhan, China; Department of Cardiology, Union Hospital, Tongji Medical College, Huazhong University of Science and Technology, Wuhan, China; Department of Rheumatology, Union Hospital, Tongji Medical College, Huazhong University of Science and Technology, Wuhan, China

**Keywords:** Autoantibody, Autoimmune disease, Genetic predisposition, Single-nucleotide polymorphism

## Abstract

rs2431697 is located on 5q33.3, between pituitary tumor-transforming gene 1 and miR-146a. Several studies have estimated the association between rs2431697 and systemic lupus erythematosus risk. However, the results were inconsistent. A case-control study was carried out to explore the association between rs2431697 and systemic lupus erythematosus risk in a central Chinese population. Meta-analyses combining present with previous studies were conducted to further explore the association. Our case-control study included 322 cases and 353 controls. rs2431697 T allele was associated with increased risk of systemic lupus erythematosus (odds ratios (ORs) = 1.461, 95 % confidence intervals (CI) 1.091–1.957, *P* = 0.011). The association was stronger between T allele and the risk of anti-double-stranded DNA (dsDNA)-positive systemic lupus erythematosus (OR = 2.510, 95 % CI 1.545–4.077, *P* < 0.001). The meta-analyses included 8648 systemic lupus erythematosus patients and 10947 controls. rs2431697 T allele had an overall OR of 1.262 (95 % CI 1.205–1.323, *P* < 0.001) under fixed-effects model. After stratified by ethnicity, *I*^2^ reduced from 24.3 to 0 %. T allele had an OR of 1.213 (95 % CI 1.145–1.284, *P* < 0.001) in European descendant and 1.365 (95 % CI 1.259–1.480, *P* < 0.001) in Asian under fixed-effects model. Data on women were also extracted, and T allele had an OR of 1.337 (95 % CI 1.162–1.539, *P* < 0.001) under random-effects model. The pooled ORs were not influenced by each study in sensitivity analyses. There were no publication biases observed in these analyses. The results from our case-control study and the meta-analyses indicate that rs2431697 T allele significantly associates with the increased risk of systemic lupus erythematosus.

**Electronic supplementary material:**

The online version of this article (doi:10.1007/s10067-015-3045-4) contains supplementary material, which is available to authorized users.

## Introduction

Systemic lupus erythematosus (SLE, Online Mendelian Inheritance in Man [OMIM] 152700) is a prototypic autoimmune disease characterized by autoantibody production (such as anti-double-stranded DNA (dsDNA)), immune complex deposition, and tissue destruction. SLE mainly affects women, especially during childbearing years [1, 2]. Studies based on familial members found a high sibling risk ratio (*λ*_sibling_ = 5.8–29) [3] and the concordance rate in monozygotic twins to be about ten times higher than in dizygotic twins [4]. Many evidences demonstrated that SLE is deeply influenced by genetic factors [5].

Recent genome-wide association studies (GWAS) have provided us many SLE susceptibility single-nucleotide polymorphisms (SNPs). Besides those classic immunology molecule-associated SNPs, there are also many other new SNPs [5]. rs2431697 is one of these newly discovered SNPs [5, 6]. rs2431697 is located on chromosome 5q33.3, between pituitary tumor-transforming gene 1 (PTTG1) and miR-146. Currently, the biological function of this SNP is not clear. Most of rs2431697-related studies are focused on exploring gene frequency in different populations. Two studies reported that rs2431697 was associated with overall SLE risk in European descendant [7, 8]. However, Chung et al. found that rs2431697 was not associated with anti-dsDNA-negative SLE risk in European descendant [9]. Two studies reported that rs2431697 was associated with female SLE risk in European descendant and Korea population [6, 10], but this association was not proved in a Chinese population [11]. Since the relationship between rs2431697 and the risk of SLE is inconsistent, more studies, especially based on more detailed subgroup populations, are needed to amplify the data. In this study, we carried out an additional case-control study on the relationship between rs2431697 and SLE risk in a central Chinese population and presented a meta-analysis based on currently available data. We hope to clarify the association between rs2431697 and SLE risk.

## Materials and methods

### Case-control study sample

SLE sample composed of 322 unrelated Han Chinese individuals (288 women, 34 men) from Hubei Province, China. The mean age was 36 (25–75 %, 24–44; youngest to oldest, 12–69). All the patients fulfilled the SLE diagnosis criteria of American College of Rheumatology (1997). SLE-associated autoantibodies (anti-dsDNA, anti-sm) were detected by Western blot/EUROLINE-WB Test Systems (EUROIMMUN, Germany) in the department of Laboratory Medicine, Union Hospital, Tongji Medical College. Healthy Han Chinese included 353 individuals (322 women, 31 men) recruited from the Medical Examination Center, Union Hospital. The mean age was 39 (25–75 %, 27–56; youngest to oldest, 22–65). All the healthy individuals had no evidence of SLE or other autoimmune diseases. All individuals gave informed consent. The study was carried out following the Declaration of Helsinki and approved by the Ethics Committee of Union Hospital, Tongji Medical College, Huazhong University of Science and Technology.

### Genotyping

Genomic DNA was extracted and purified from peripheral blood with DNA Extractor WB Kit (Wako Pure Chemical Industries, Ltd. Japan) according to the product description. rs2431697 polymorphism was determined based on polymerase chain reaction-restriction fragment length polymorphism (PCR-RFLP) assay. A 260-bp DNA fragment around rs2431697 was amplified with the primer pair: sense primer: 5′-AGAGGGGGTGAAAGAAGGAA-3′ and antisense primer: 5′-TTCTCAGTGCCAATGTGAGG-3′. The reaction mixtures contained 10 ng genomic DNA, 10 pmol of each primer, and double-diluted Taq 2× Master Mix (New England Biolabs, UK) in a total 20-μl volume. The reactions were carried out in a T3 thermocycler (Biometra, Göttingen, Germany). The reaction conditions were as follows: denaturation at 94 °C for 5 min, followed by 35ccycles of denaturation at 94 °C for 30 s, annealing for 1 min at 57 °C and extension at 72 °C for 45 s, and a final extension at 72 °C for 5 min. PCR products were subsequently digested by Taq I (New England Biolabs, UK) at 65 °C for 2 h and separated on a 3 % agarose gel. The rs2431697 T allele yielded 61- and 199-bp fragments, and C allele yielded a single 260-bp fragment.

### Statistical analysis

Hardy-Weinberg equilibrium was analyzed by the chi-square goodness of fit test for genotypes in the control group. The differences of allele and genotype frequencies between SLE patients and health controls were compared by the chi-square test. Unconditional multivariate logistic regression analysis was used to estimate odds ratios (ORs) and 95 % confidence intervals (CI) for the effect of rs2431697 on SLE risk, adjusted for age and sex. PS: Power and Sample Size Calculation version 3.0 [12] was used to calculate the power on OR value based on the number of subjects. The statistical significance was defined as *P* (two-tailed) <0.05. All analyses were performed by SPSS (Version 12.0).

### Meta-analysis

To further estimate the association between rs2431697 and the risk of SLE, a meta-analysis according to the guideline of Preferred Reporting Items for Systemic Reviews and Meta-Analysis (PRISMA) statement was performed. Studies were searched and retrieved through PUBMED and EMBASE without language restriction. The search keywords were “rs2431697,” “miR-146a,” or “pttg1” or “pituitary tumor-transforming protein 1” in combination with “systemic lupus erythematosus”. Results were limited in studies of humans and not experiments designed. The reference lists of included articles and related review articles were manually searched. The final search was carried out on 8 December 2014. Retrieved studies were restricted in publication type of article or article in press and eligible if they were case-control designed and if they provided (in the primary paper or by email request) the frequencies of alleles and/or genotypes for rs2431697 in SLE cases and healthy controls. Data that overlapped with others was excluded. Two authors (ZMT, PW) independently read and extracted data. A third author helped to decide if controversy occurred. The following information was extracted: name of first author, year of publication, ethnicity, matching variable, sizes of cases and controls, numbers or frequencies of alleles, or genotypes in cases and controls.

For each included study, Hardy-Weinberg equilibrium in controls was deduced or extracted from the primary papers. Pooled ORs and 95 % CIs of the T allele in overall populations, ethnicity-stratified populations, and female populations were estimated. Pooled ORs and 95 % CIs of rs2431697 genotypes, dominant genetic model, and recessive genetic model in overall populations and female populations were also assumed.

Heterogeneity of the eligible studies was assessed by Cochran’s *Q* test and *I*^2^ value. Random-effects model was used if the *P* value of *Q* test was less than 0.1 or *I*^2^ value was larger than 50 %. Otherwise, fixed-effects model was carried out. Sensitivity analysis was performed to assess the influence of each study. Publication bias was estimated by funnel plot [13] and Egger’s test [14]. All statistical analyses were performed by STATA V11.2

## Results

### Case-control study

The clinical and immunological characteristics of the included patients and the controls are presented in Table 1. The genotype and allele distributions of rs2431697 are presented in Table 2. The distribution of genotypes in healthy controls was not deviated from Hardy-Weinberg equilibrium (*P* = 0.8041). The distribution of rs2431697 T allele was significantly increased in SLE patients compared with controls (OR = 1.461, 95 % CI 1.091–1.957, *P* = 0.011). There were no differences found in genotype distribution between SLE patients and controls, except for the recessive model (OR = 1.521, 95 % CI 1.091–2.120, *P* = 0.013) (Table 2).

**Table 1:** Clinical characteristics of the participants

	SLE	Healthy controls
No. of cases	322	353
No. men/no. women	34/288	31/322
Age, median (range) (years)	36 (12–69)	39 (22–65)
Disease manifestations, number (%)		
Lupus nephritis	200 (62)	0
Vasculitis	81 (25)	0
Arthritis	97 (30)	0
Rash	32 (10)	0
Alopecia	48 (15)	0
Mucosal ulcers	74 (23)	0
Serositis	55 (17)	0
Leukopenia	103 (32)	0
Thrombocytopenia	48 (15)	0
Fever	61 (19)	0
Visual disturbance	0	0
Anti-dsDNA positive, number (%)	129 (40)	0
Anti-sm-positive number (%)	123 (38)	0
SLEDAI	8 (0–25)	0
Proteinuria (mg/24 h)	271 (45–8852)	N/A
Serum creatinine (μmol/L)	53.9 (24.4–495.6)	65.3 (42.3–134.2)
C3 (g/L)	0.64 (0.12–11.82)	1.08 (0.53–6.64)
C4 (g/L)	0.12 (0.02–0.42)	0.28 (0.15–0.52)

**Table 2:** The association between rs2431697 and SLE risk in a Chinese population

		All samples				Females			
		SLE (fre.)	Con. (fre.)	OR (95 % CI)	*P* value	SLE (fre.)	Con. (fre.)	OR (95 % CI)	*P* value
Genotype	TT	239 (0.74)	231 (0.65)			216 (0.75)	209 (0.65)		
	TC	77 (0.24)	110 (0.31)			66 (0.23)	101 (0.31)		
	CC	6 (0.02)	12 (0.03)			6 (0.02)	12 (0.04)		
	TT vs CC			2.069 (0.764, 5.605)	0.145			2.067 (0.762, 5.609)	0.146
	TC vs CC			1.418 (0.510, 3.941)	0.501			1.307 (0.468, 3.653)	0.609
	Dominant model			1.853 (0.687, 4.997)	0.216			1.819 (0.674, 4.912)	0.231
	Recessive model			1.521 (1.091, 2.120)	0.013			1.622 (1.141, 2.305)	0.007
Allele	T	555 (0.86)	572 (0.81)			498 (0.86)	519 (0.81)		
	C	89 (0.14)	134 (0.19)			78 (0.14)	125 (0.19)		
	T vs C			1.461 (1.091, 1.957)	0.011			1.538 (1.130, 2.093)	0.006

*Con*. control, *fre*. frequency

Considering the gender disposition of SLE, data on women were extracted for separated analysis. Significant statistical difference was seen in T allele frequencies between SLE patients and healthy controls (OR = 1.538, 95 % CI 1.130–2.093, *P* = 0.006) (Table 2). Statistically significant difference in recessive model between SLE patients and healthy controls was also found (OR = 1.622, 95 % CI 1.141–2.305, *P* = 0.007).

Anti-dsDNA and anti-sm are SLE-specific autoantibodies. SLE patients were subgrouped by the existence or inexistence of anti-dsDNA and anti-sm autoantibodies. There were 123 anti-dsDNA-positive and 171 anti-dsDNA-negative individuals included in this case-control study. The frequency of rs2431697 T allele in anti-dsDNA-positive patients was 0.91. The T allele was substantially increased in anti-dsDNA-positive patients compared with healthy controls (OR = 2.510, 95 % CI 1.545–4.077, *P* < 0.001). In anti-dsDNA-negative patients, rs2431697 T allele frequency was 0.84; there was no statistic difference between SLE patients and healthy controls (OR = 1.196, 95 % CI 0.849–1.685, *P* = 0.305). Similar results were also observed when data were subgrouped by anti-sm condition.

### Meta-analysis

#### Study characteristics

**Fig. 1:**
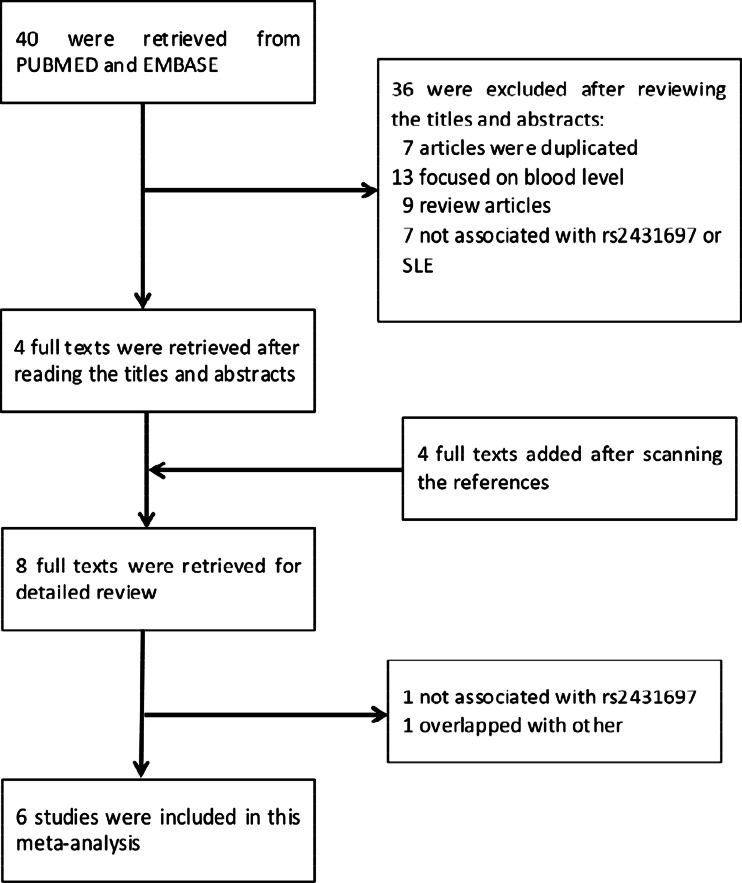
Flow chart of the study selection process

**Fig. 2:**
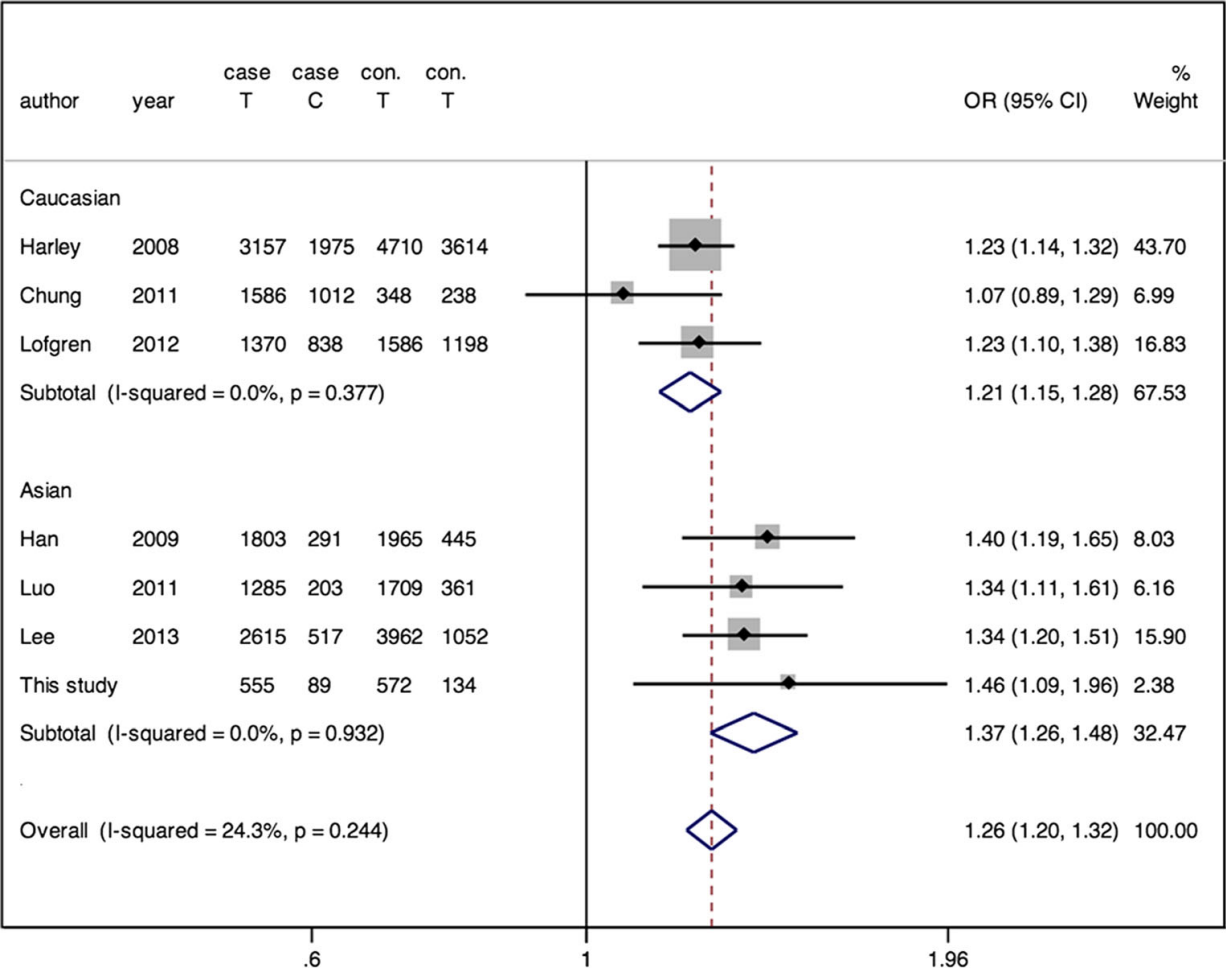
Forest plot of association between rs2431697 and SLE risk under allelic model in the meta-analysis

**Fig. 3:**
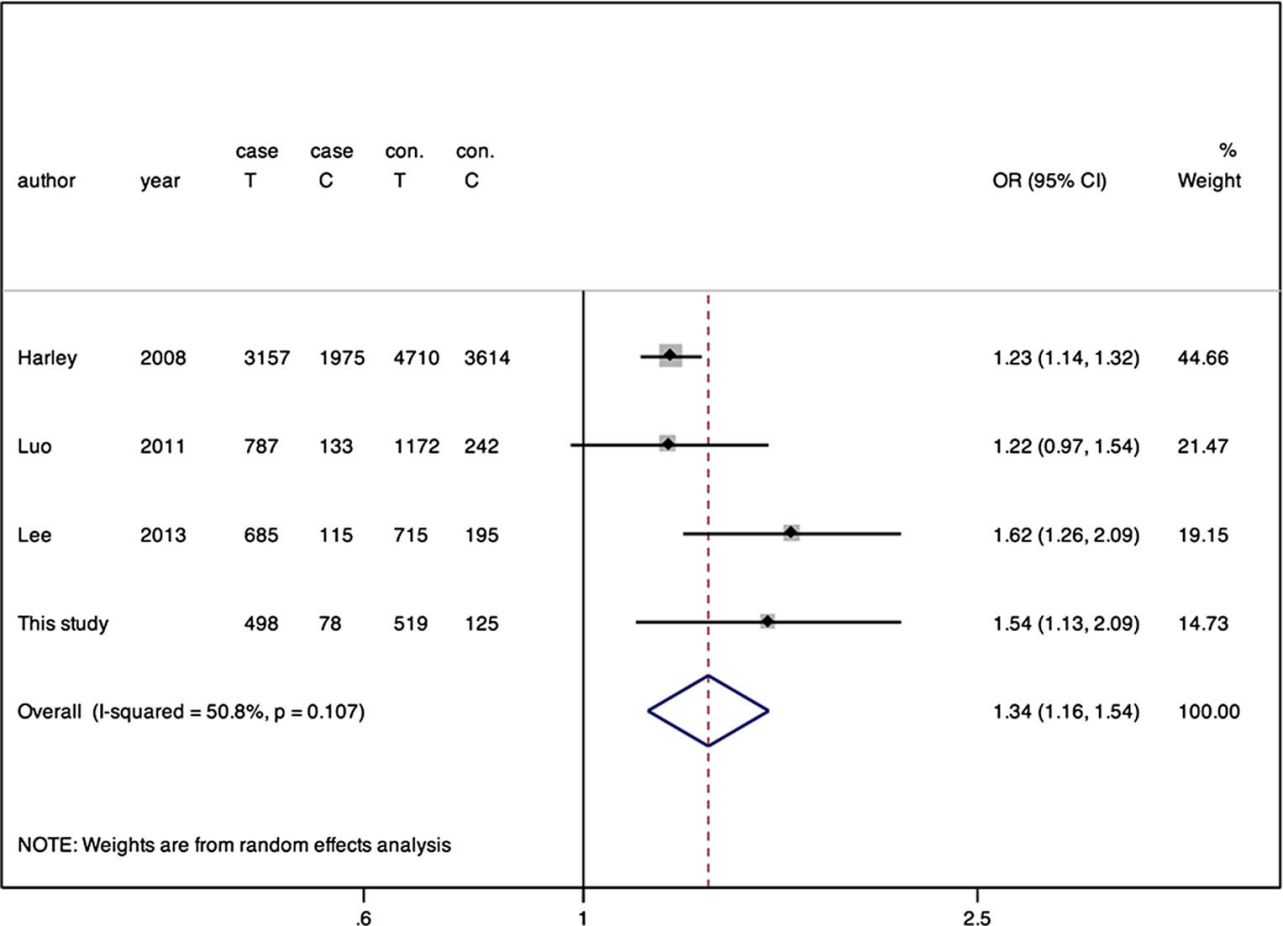
Forest plot of association between rs2431697 and SLE risk under allelic model in the women data-based meta-analysis

There were 40 studies identified from PUBMED and EMBASE. Another four studies were also added for review [6, 7, 9, 15] by manually retrieving the references. After detailed consulting the author, a segment of control data in the paper of Chung [9] was not included because the populations overlapped with the study of Harley et al. [6]. Finally, as the flow chart shown in Fig. 1, there were seven studies included in this meta-analysis including our case-control study. A study by Ramos et al. [7] was not included because the population overlapped with the study by Harley et al. [6]. Among the included studies, three were conducted in European descendant populations, two were conducted in Chinese, and one was conducted in Korean. The meta-analysis totally contained 8648 SLE subjects and 10,947 controls. Genotypes in all control groups were in Hardy-Weinberg equilibrium according to the direct mention in the studies or indirect calculating. The distributions of rs2431697 alleles and genotypes are shown in the forest plots, respectively (Figs. 2 and 3, Online Resource 1, Online Resource 2).

#### Overall meta-analysis of the association between rs2431697 and SLE

There was no obvious heterogeneity observed in different kinds of genetic models. Fixed-effects model was applied in these analyses. Overall, there was a statistically significant relation between rs2431697 T allele and the increased SLE risk (OR = 1.262, 95 % CI = 1.205–1.323; *Z* = 9.78, *P* < 0.001) (Fig. 2). In the genotype model, statistically significant increase of SLE risk was observed for TT versus CC (OR = 1.453, *P* < 0.001), but not CT versus CC (OR = 1.117, *P* = 0.182). Increased SLE risk was also observed in dominant and recessive models (*P* < 0.05, Online Resource 1).

#### Stratified meta-analysis of the association between rs2431697 and SLE

The prevalent rates of SLE vary in different ethnicities. To explore if the association between rs2431697 T allele and SLE risk was influenced by ethnical factor, data were separated by ethnicity. After separating, the heterogeneity among the included studies greatly decreased. The value of *I*^2^ decreased from 24.3 to 0.0 %. The pooled OR was 1.213 (95 % CI 1.145–1.284, *P* < 0.001) in European descendant and 1.365 (95 % CI 1.259–1.480, *P* < 0.001) in Asian (Fig. 2).

Considering that gender is a predisposed factor of SLE, data specific to women were separated for analysis. Men-specific data were not grouped because of limited available data. There was a marginal heterogeneity among the data from women-based studies (*P* = 0.107, *I*^2^ = 50.8 %), and random-effects model was used (Fig. 3). In women, rs2431697 T allele was also associated with the risk of SLE (OR = 1.261, 95 % CI = 1.182–1.344, *P* < 0.001) (Fig. 3). The pooled ORs for genotypic models are shown in Online Resource 2.

#### Sensitivity analysis and publication bias

**Fig. 4:**
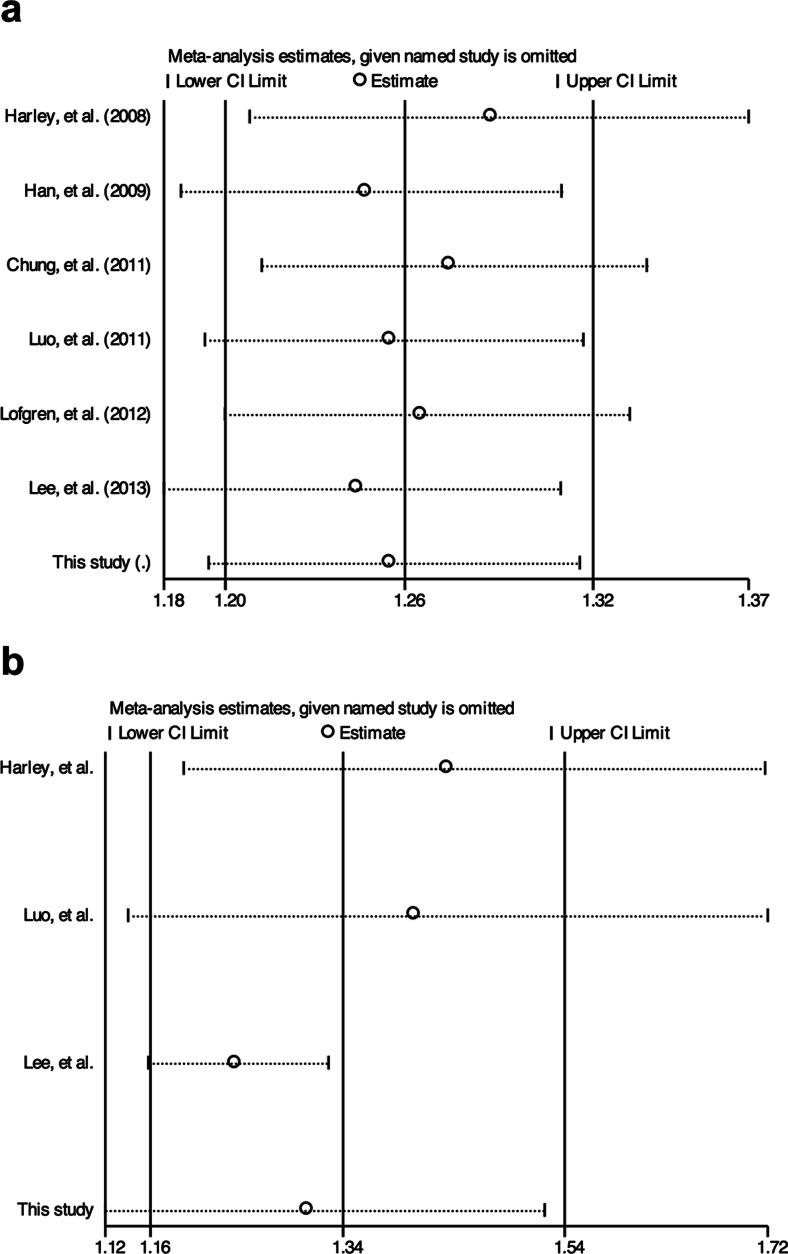
Sensitivity analysis of allelic model for overall or women-only meta-analysis. **a** Overall meta-analysis. **b** Women data-based meta-analysis

Sensitivity analysis showed that the association of rs2431697 T allele with SLE remained significant after removing any one study in the overall and women-only meta-analysis (Fig. 4). Sensitivity analysis also found that no single study can substantially change the pooled ORs of other data set (data not shown).

Egger’s test and Funnel plots were carried out to estimate the publication bias. For each data set, the Egger’s test results are provided in Table 3. There was no publication bias observed in each data set analysis. Funnel plots showed similar results as Egger’s test.

**Table 3:** Egger’s test results for publication bias of allelic model and genotypic models

Comparisons	*Y* axis intercept *P* value (95 % CI)				
	T vs C	TT vs CC	TC vs CC	Dominant model	Recessive model
Overall	0.438 (−1.926, 3.804)	0.873 (−5.321, 4.771)	0.741 (−5.003, 3.980)	0.944 (−4.559, 4.781)	0.845 (−5.603, 4.901)
European descendant	0.398 (−18.896,15.181)				
Asian	0.297 (−1.614, 3.168)				
Women	0.232 (−3.126, 7.197)	0.462 (−126.739, 151.424)	0.484 (−128.446, 151.661)	0.462 (−126.493, 151.113)	0.551 (−39.872, 45.594)

## Discussion

Benefitting from the technology of GWAS, large numbers of SLE-associated SNPs were discovered [5]. The SNP rs2431697 was involved in some SLE-related GWAS and replication studies, but the results were inconsistent. The present case-control study demonstrates a significant association between rs2431697 T allele and the risk of SLE in the population from central China (OR = 1.461, *P* = 0.011). We further confirm this association with a meta-analysis that includes 8648 SLE patients and 10,947 controls (OR = 1.262, *P* < 0.001). The current data also suggest that association between rs2431697 and SLE risk is under recessive model and related with autoantibody-positive condition.

The SNP rs2431697 is located in an intergenic region with 24.23 kb from downstream of PTTG1 gene and 15.3 kb from upstream of miR-146a [6, 16]. Currently, there is no direct evidence to confirm which gene that this SNP is related to. Lofgren et al. found that rs2431697 T allele was associated with downregulated expression of miR-146a but not PTTG1 in peripheral blood mononuclear cells obtained from Europeans [16]. Bioinformatics method suggested that rs2431697 locates in a high potential regulatory region of miR-146a [16]. MiR-146a is one of the initially appreciated SLE-related microRNAs (miRNAs) [17]. This miRNA can repress type 1 interferon (IFN) pathway through targeting TNF receptor-associated factor 6, IL-1 receptor-associated kinase, IFN regulator factor 5 (IRF5), and STAT-1 [17–19]. In the development process of SLE, the enhanced type 1 IFN signal pathway plays a critical role [20–22]. Thus, T allele of rs2431697 may contribute to the pathological process of SLE by downregulating the expression of miR-146a in Europeans [16]. In the present case-control study, our data also suggests the relationship between rs2431697 T allele and SLE risk in a population from central China. The present meta-analysis results suggest that T allele and TT genotype are significantly associated with SLE risk totally at the population level. Currently, there is no direct experiment to explain this relationship. Contrary to the finding of Lofgren [16], Luo et al. did not find the relationship between rs2431697 and miR-146a level [11]. The difference may be caused by the following reasons. Firstly, in the study of Luo, miRNA was purified from leucocytes that include granulocyte, lymphocyte, and monocyte, while granulocyte was not included in the study of Lofgren. Different miRNA sources may contribute to the different results between the two studies; this cannot be ruled out, since there is no study to explore the expression profile in granulocyte from SLE patients. Secondly, different SLE activity in these two groups may contribute to the different results; however, the two studies did not provide the information on SLE activity. Thirdly, different populations between these two studies may contribute to the different results. We are performing experiments to explore the relationship between rs2431697 and the circulating level of miR-146a in central Chinese population.

The effect size was slightly higher in our case-control study. Our SLE patients were recruited from rheumatology inpatient department in a large metropolitan health center. A big proportion of inpatients entering this kind of health center in China is from rural areas and has more severe disease conditions. Our cohort may be biased toward the more severe patients. However, the association between rs2431697 and disease severity is yet to be evaluated.

A previous study has shown that some gene polymorphisms were specifically associated with the risk of autoantibody-positive rheumatoid arthritis [23]. Since the function of rs2431697 is largely unknown, exploring the association between rs2431697 and the production of antibodies may help to improve the understanding of its functions in SLE pathophysiology. The production of autoantibodies, such as anti-dsDNA, is an important pathological characteristic of SLE [24, 25]. Currently, only one study examined the relationship between anti-dsDNA condition and rs2431697 in SLE patients [9]. Chung et al. found that the association between rs2431697 T allele and the risk of SLE occurred in anti-dsDNA positive but not negative patients of European descent [9]. This finding was replicated in our case-control study based on central Chinese population. Our data further suggest that rs2431697 is also associated with anti-sm-positive SLE risk. These data imply that rs2431697 is associated with the production of autoantibodies in SLE patients.

The prevalence of SLE was different across different populations [26]. Both environmental factors and the difference of genetic background are major determinants [26]. Yang et al. found that Chinese living in Hong Kong, Taiwan, and Beijing have different spectrum of SLE risk alleles [27]. In the present case-control study, in respect to rs2431697 allele frequency, we did not find differences between the population from Hubei Province and other Chinese mainland population both in health control group and in SLE group [15]. In our meta-analysis, data were stratified into European descendant group and Asian group. After stratifying, moderate heterogeneity disappeared (*I*^2^ from 24 to 0 %). It suggests that ethnicity is the main source of heterogeneity. The pooled OR strongly indicated that the T allele of rs2431697 is an SLE risk factor both in Asian and in European descendant. Studies based on more ethnic populations are needed to improve this result.

The T allele of rs2431697 was first suggested to be associated with SLE susceptibility in women [6]. In consideration of gender factor in the onset and development of SLE [28], female participants were stratified from the total participants in this study. From our case-control results, no obvious difference was found on the association between rs2431697 and SLE risk when comparing gender-combined population (OR 1.461, 95 % CI 1.091–1.957) with female population (OR 1.538, 95 % CI 1.130–2.093). The difference was even smaller in the meta-analysis results (gender-combined, OR 1.262, 95 % CI 1.205–1.323; female, OR 1.260, 95 % CI 1.180–1.340). The data on men were not stratified for analysis because the data was limited. While men among SLE patients often face a more severe experience [28] and genetic background influence more profound in men than women in the development of SLE [29]. Hughes et al. found that men with SLE possess a higher frequency of the risk allele of IRF5 than women [29]. IRF5 is included in the signal pathway of miR-146a as mentioned above. From this point, more data on men are needed to determine if the association between rs2431697 and SLE risk can be influenced by gender.

There are some limitations in this study. Firstly, the sample size of our case-control study is not large enough, resulting in moderate statistical power (0.724). However, the allele frequency in our study is similar to another large Chinese population-based results [15] and also that reported in HapMap. Secondly, for meta-analysis, the number of included papers is small, and the pooled results can be influenced by the large sample size-designed study. Thirdly, concerning GWAS, only those with positive relation were included, while those with negative relation [27, 30] were not included because of unavailable data. This could be a potential cause of selection bias. Fourthly, available data could not support us to perform meta-analysis stratified by age, while a study has found that SLE susceptibility genes were influenced by disease onset age [31]. During the preparing of this paper, Ji et al. reported a meta-analysis on the relationship between rs2431697 and SLE risk based on only two studies [32]. Our study increased the data much more.

## Conclusions

In conclusion, this study adds to the growing evidence of the effect of rs2431697 on SLE risk. Our meta-analysis verified a significant association between rs2431697 T allele and SLE risk in Asian and European descendant populations. Our case-control study also indicated that rs2431697 T allele was associated with the risk of SLE with anti-dsDNA positive but not negative condition. Experimental data are expected to directly demonstrate the pathophysiological mechanism of rs2431697 in SLE risk.

## Electronic supplementary material

Online Resource 1 (PDF 187 kb)

Online Resource 2 (PDF 176 kb)
