# Supplementary material for: Association between rs2431697 T allele on 5q33.3 and systemic lupus erythematosus: case-control study and meta-analysis
Source: Clin Rheumatol. 2015 Aug 7;34(11):1893–902. doi: 10.1007/s10067-015-3045-4 (PMC4624827; doi:10.1007/s10067-015-3045-4)
Supplement: Supplementary file 1 — (PDF 187 kb) [file 10067_2015_3045_MOESM1_ESM.pdf]

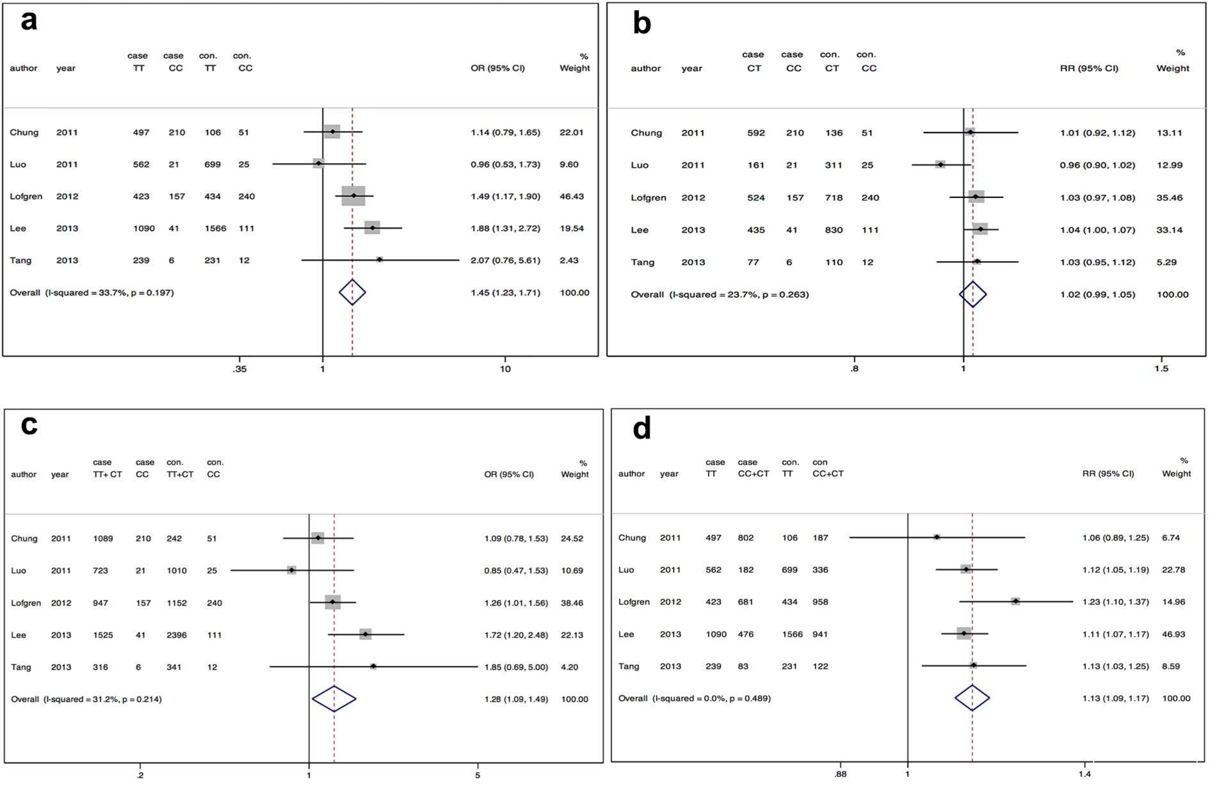
a
author year case TT case CC con. TT con. CC OR (95% CI) % Weight
Chung 2011 497 210 106 51 1.14 (0.79, 1.65) 22.01
Luo 2011 562 21 699 25 0.96 (0.53, 1.73) 9.60
Lofgren 2012 423 157 434 240 1.49 (1.17, 1.90) 46.43
Lee 2013 1090 41 1566 111 1.88 (1.31, 2.72) 19.54
Tang 2013 239 6 231 12 2.07 (0.76, 5.61) 2.43
Overall (I-squared = 33.7%, p = 0.197) 1.45 (1.23, 1.71) 100.00
.35
1
10
b
author year case CT case CC con. CT con. CC RR (95% CI) % Weight
Chung 2011 592 210 136 51 1.01 (0.92, 1.12) 13.11
Luo 2011 161 21 311 25 0.96 (0.90, 1.02) 12.99
Lofgren 2012 524 157 718 240 1.03 (0.97, 1.08) 35.46
Lee 2013 435 41 830 111 1.04 (1.00, 1.07) 33.14
Tang 2013 77 6 110 12 1.03 (0.95, 1.12) 5.29
Overall (I-squared = 23.7%, p = 0.263) 1.02 (0.99, 1.05) 100.00
.8
1
1.5
c
author year case TT+ CT case CC con. TT+CT con. CC OR (95% CI) % Weight
Chung 2011 1089 210 242 51 1.09 (0.78, 1.53) 24.52
Luo 2011 723 21 1010 25 0.85 (0.47, 1.53) 10.69
Lofgren 2012 947 157 1152 240 1.26 (1.01, 1.56) 38.46
Lee 2013 1525 41 2396 111 1.72 (1.20, 2.48) 22.13
Tang 2013 316 6 341 12 1.85 (0.69, 5.00) 4.20
Overall (I-squared = 31.2%, p = 0.214) 1.28 (1.09, 1.49) 100.00
.2
1
5
d
author year case TT case CC+CT con. TT con CC+CT RR (95% CI) % Weight
Chung 2011 497 802 106 187 1.06 (0.89, 1.25) 6.74
Luo 2011 562 182 699 336 1.12 (1.05, 1.19) 22.78
Lofgren 2012 423 681 434 958 1.23 (1.10, 1.37) 14.96
Lee 2013 1090 476 1566 941 1.11 (1.07, 1.17) 46.93
Tang 2013 239 83 231 122 1.13 (1.03, 1.25) 8.59
Overall (I-squared = 0.0%, p = 0.489) 1.13 (1.09, 1.17) 100.00
.88
1
1.4

Online Resource 1 Forest plot of association between rs2431697 and SLE risk under genotypic model in the meta-analysis a: TT vs CC b: CT vs CC c: TT+CT vs CC d: TT vs CC+CT

Title: Association between rs2431697 T allele on 5q33.3 and systemic lupus erythematosus: case-control study and meta-analysis

Journal name: Clinical Rheumatology

Author: Zhao-Ming Tang

Affiliation: Department of Laboratory Medicine, Union Hospital, Tongji Medical College, Huazhong University of Science and Technology, Wuhan, China

Author: Ping Wang

Affiliation: Department of Laboratory Medicine, Union Hospital, Tongji Medical College, Huazhong University of Science and Technology, Wuhan, China

Author: Pan-Pan Chang

Affiliation: Central Laboratory, Union Hospital, Tongji Medical College, Huazhong University of Science and Technology, Wuhan, China

Author: Tony Hasahya

Affiliation: Department of Cardiology, Union Hospital, Tongji Medical College, Huazhong University of Science and Technology, Wuhan, China

Author: Hui Xing

Affiliation: Department of Laboratory Medicine, Union Hospital, Tongji Medical College, Huazhong University of Science and Technology, Wuhan, China

Author: Jin-Ping Wang

Affiliation: Department of Rheumatology, Union Hospital, Tongji Medical College, Huazhong

University of Science and Technology, Wuhan, China

Author: Li-Hua Hu (Corresponding author)

Affiliation: Department of Laboratory Medicine, Union Hospital, Tongji Medical College, Huazhong University of Science and Technology, Wuhan, China

Email: toumin@126.com
