# Supplementary material for: Association between rs2431697 T allele on 5q33.3 and systemic lupus erythematosus: case-control study and meta-analysis
Source: Clin Rheumatol. 2015 Aug 7;34(11):1893–902. doi: 10.1007/s10067-015-3045-4 (PMC4624827; doi:10.1007/s10067-015-3045-4)
Supplement: Supplementary file 2 — (PDF 176 kb) [file 10067_2015_3045_MOESM2_ESM.pdf]

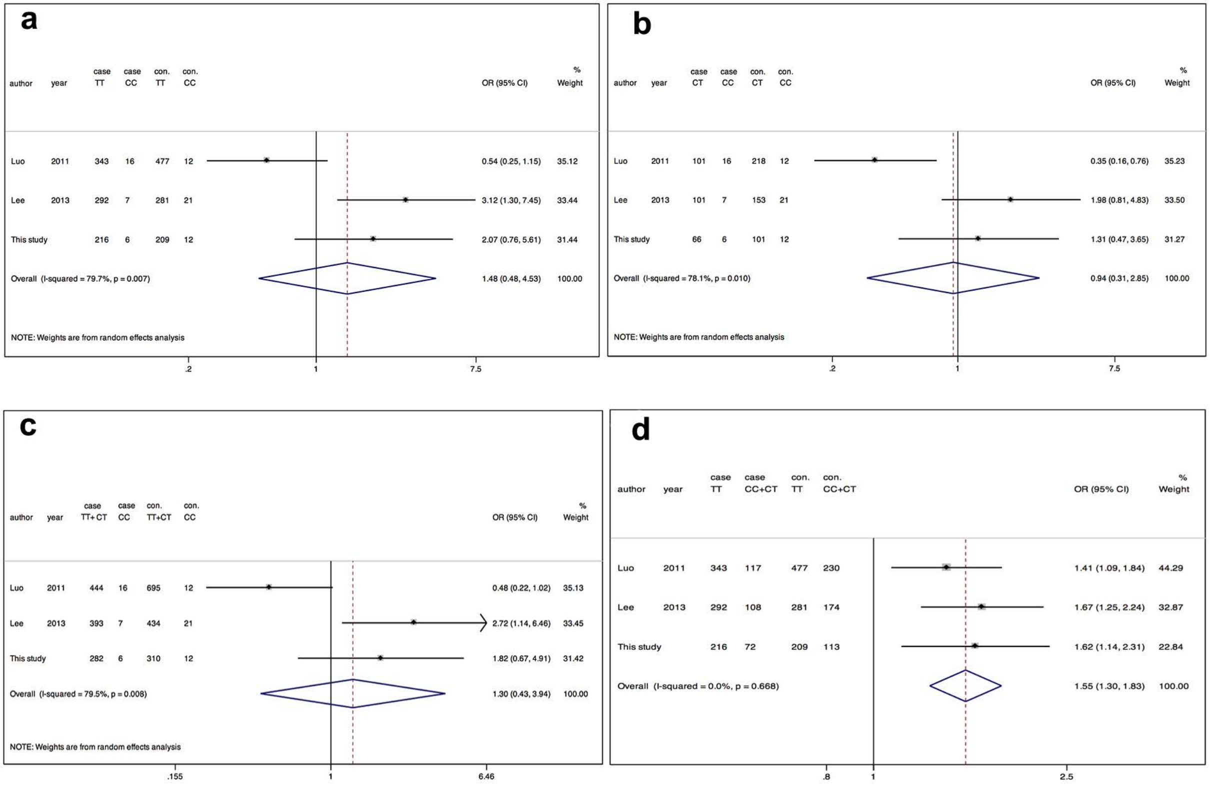
a
author year case TT case CC con. TT con. CC OR (95% CI) % Weight
Luo 2011 343 16 477 12 0.54 (0.25, 1.15) 35.12
Lee 2013 292 7 281 21 3.12 (1.30, 7.45) 33.44
This study 216 6 209 12 2.07 (0.76, 5.61) 31.44
Overall (I-squared = 79.7%, p = 0.007) 1.48 (0.48, 4.53) 100.00
NOTE: Weights are from random effects analysis
.2
1
7.5
b
author year case CT case CC con. CT con. CC OR (95% CI) % Weight
Luo 2011 101 16 218 12 0.35 (0.16, 0.76) 35.23
Lee 2013 101 7 153 21 1.98 (0.81, 4.83) 33.50
This study 66 6 101 12 1.31 (0.47, 3.65) 31.27
Overall (I-squared = 78.1%, p = 0.010) 0.94 (0.31, 2.85) 100.00
NOTE: Weights are from random effects analysis
.2
1
7.5
c
author year case TT+CT case CC con. TT+CT con. CC OR (95% CI) % Weight
Luo 2011 444 16 695 12 0.48 (0.22, 1.02) 35.13
Lee 2013 393 7 434 21 2.72 (1.14, 6.46) 33.45
This study 282 6 310 12 1.82 (0.67, 4.91) 31.42
Overall (I-squared = 79.5%, p = 0.008) 1.30 (0.43, 3.94) 100.00
NOTE: Weights are from random effects analysis
.155
1
6.46
d
author year case TT case CC+CT con. TT con. CC+CT OR (95% CI) % Weight
Luo 2011 343 117 477 230 1.41 (1.09, 1.84) 44.29
Lee 2013 292 108 281 174 1.67 (1.25, 2.24) 32.87
This study 216 72 209 113 1.62 (1.14, 2.31) 22.84
Overall (I-squared = 0.0%, p = 0.668) 1.55 (1.30, 1.83) 100.00
.8
1
2.5

Online Resource 2 Forest plot of association between rs2431697 and SLE risk under genotypic model in the women data-based meta-analysis a: TT vs CC b: CT vs CC c: TT+CT vs CC d: TT vs CC+CT

Title: Association between rs2431697 T allele on 5q33.3 and systemic lupus erythematosus: case-control study and meta-analysis

Journal name: Clinical Rheumatology

Author: Zhao-Ming Tang

Affiliation: Department of Laboratory Medicine, Union Hospital, Tongji Medical College, Huazhong University of Science and Technology, Wuhan, China

Author: Ping Wang

Affiliation: Department of Laboratory Medicine, Union Hospital, Tongji Medical College, Huazhong University of Science and Technology, Wuhan, China

Author: Pan-Pan Chang

Affiliation: Central Laboratory, Union Hospital, Tongji Medical College, Huazhong University of Science and Technology, Wuhan, China

Author: Tony Hasahya

Affiliation: Department of Cardiology, Union Hospital, Tongji Medical College, Huazhong University of Science and Technology, Wuhan, China

Author: Hui Xing

Affiliation: Department of Laboratory Medicine, Union Hospital, Tongji Medical College, Huazhong University of Science and Technology, Wuhan, China

Author: Jin-Ping Wang

Affiliation: Department of Rheumatology, Union Hospital, Tongji Medical College, Huazhong University of Science and Technology, Wuhan, China

Author: Li-Hua Hu (Corresponding author)

Affiliation: Department of Laboratory Medicine, Union Hospital, Tongji Medical College, Huazhong University of Science and Technology, Wuhan, China

Email: toumin@126.com
